# Cost of hospital services in India: a multi-site study to inform provider payment rates and Health Technology Assessment

**DOI:** 10.1186/s12913-022-08707-7

**Published:** 2022-11-14

**Authors:** Akashdeep Singh Chauhan, Lorna Guinness, Pankaj Bahuguna, Maninder Pal Singh, Vipul Aggarwal, Kavitha Rajsekhar, Surbhi Tripathi, Shankar Prinja

**Affiliations:** 1 Department of Community Medicine and School of Public Health, Post Graduate Institute of Medical Education and Research, Chandigarh, India; 2 King’s Technology Evaluation Centre, King’s College, London, UK; 3 Centre for Global Development, Great Peter House, Great College St, Abbey Gardens, London, SW1P 3SE UK; 4 Department of Global Health and Development, London School of Hygiene and Tropical Medicine, London, UK; 5 School of Health and Wellbeing, College of Medical, Veterinary and Life Sciences, Health Economics and Health Technology Assessment, University of Glasgow, Glasgow, UK; 6 National Health Authority, Government of India, New Delhi, India; 7 Department of Health Research, Government of India, New Delhi, India

**Keywords:** Costing, Hospitals, India, Efficiency, Private providers, Prices

## Abstract

The 'Cost of Health Services in India (CHSI)' is the first large scale multi-site facility costing study to incorporate evidence from a national sample of both private and public sectors at different levels of the health system in India. This paper provides an overview of the extent of heterogeneity in costs caused by various supply-side factors.

A total of 38 public (11 tertiary care and 27 secondary care) and 16 private hospitals were sampled from 11 states of India. From the sampled facilities, a total of 327 specialties were included, with 48, 79 and 200 specialties covered in tertiary, private and district hospitals respectively. A mixed methodology consisting of both bottom-up and top-down costing was used for data collection. Unit costs per service output were calculated at the cost centre level (outpatient, inpatient, operating theatre, and ICU) and compared across provider type and geographical location.

The unadjusted cost per admission was highest for tertiary facilities (₹ 5690, 75 USD) followed by private facilities (₹ 4839, 64 USD) and district hospitals (₹ 3447, 45 USD). Differences in unit costs were found across types of providers, resulting from both variations in capacity utilisation, length of stay and the scale of activity. In addition, significant differences in costs were found associated with geographical location (city classification).

The reliance on cost information from single sites or small samples ignores the issue of heterogeneity driven by both demand and supply-side factors. The CHSI cost data set provides a unique insight into cost variability across different types of providers in India. The present analysis shows that both geographical location and the scale of activity are important determinants for deriving the cost of a health service and should be accounted for in healthcare decision making from budgeting to economic evaluation and price-setting.

**Supplementary Information:**

The online version contains supplementary material available at 10.1186/s12913-022-08707-7.

## Background

Recent reforms in the Indian health system with the development of the Health Technology Assessment Board (HTAIn) and the launch of the national health insurance scheme—*Ayushman Bharat Pradhan Mantri Jan Arogya Yojana* (AB PM-JAY) have highlighted the critical need for cost information on the delivery of health care services [1, 2]. While empirical evidence on the cost of health care delivery is essential for developing fair provider payment rates, it is also crucial for undertaking robust economic evaluations [3–7].

Over the last decade, there has been an increase in the availability of cost information from India. During this period, studies conducted across different states of India have generated empirical cost data on the provision of health services both at the public (inclusive of primary, secondary, and tertiary level) and private sector hospitals [8–16]. A common feature from all these studies is the extent of heterogeneity in the cost of health services delivery between similar sectors and the same level of health facilities within and across the states of India. Specifically, a study from public tertiary institutions in 11 states showed a vast difference in the cost estimates of basic services like outpatient consultation and inpatient care, even between similar specialties [12].

This cost heterogeneity is important both from the point of view of policy making and the conduct of robust research. In relation to policy-making, this heterogeneity affects the estimation of rational provider payment rates across a diverse range of providers. Under recently launched AB-PMJAY, both public (district and tertiary care facilities) and private sector hospitals have been empanelled to provide around 1600 health benefit packages (HBP) [17]. One of the core features of this scheme is the establishing of uniform reimbursement rates for the HBPs with additional subsidies for teaching hospitals, hospitals in metro cities and districts identified for support under the government’s “Aspirational District” programme [18]. This uniformity in the rates has the potential to lead to perverse incentives among the providers, for example discouraging the empanelment of smaller private hospitals that are unable to benefit from economies of scale. In addition, professional bodies, including the Indian Medical Association (IMA) & private empanelled hospitals, argue that prices of HBPs do not cover the actual cost incurred [19, 20]. The reason for dissatisfaction could be attributable to factors that cause variation in the cost of health care delivery due to demand-side (patient's perspective) factors such as the case-mix or severity of illness as well as supply-side factors including hospital ownership (public or private), level and size of the facility (secondary or tertiary, single specialty or multispecialty), scale of activity, input mix, local prices of inputs and geographical location [4, 21–24].

Secondly, the findings of economic evaluations, i.e., incremental cost-effectiveness ratio (ICER) are highly sensitive to the extent of variation in the cost parameters. The robustness of the ICER and its generalizability for a diverse nation like India depend on the cost estimates and its variation across facilities and states [3]. The growing demand for evidence in the price-setting process and economic evaluations in India highlights the need to understand the degree of heterogeneity in the cost of health care services, the reasons for such variation and the current lack of evidence in this area.

Whilst some studies describe cost estimates of health services from India, there is a dearth of evidence on the extent to which various factors influence the cost behaviour across different types of facilities in India. Implemented to inform price-setting for the AB-PMJAY and institutionalisation of Health Technology Assessment and Appraisal [3, 25] the 'Cost of Health Services in India’ (CHSI) is the first large scale multi-site facility costing study to incorporate evidence from a national sample of both private and public sectors at different levels of the health system [26]. This paper provides an overview of the extent of heterogeneity in costs caused by various supply-side factors i.e., by the type of provider, location of the hospital, efficiency, size of the facility and the scale of activity.

## Methods

### Study setting

CHSI is the first national costing study commissioned by the Department of Health Research, Government of India to generate empirical evidence on the cost of health care delivery in secondary and tertiary level health facilities [26]. The CHSI analysis specifically focussed on estimating the cost of actual resources spent by the health system in the provision of health services.

Under CHSI, a total of 38 public facilities, comprising 11 tertiary care and 27 district hospitals, and 16 private hospitals were included in the sample from 11 states of India. Out of these 11 states, four were selected from the north region (Jammu & Kashmir, New Delhi, Rajasthan and Uttar Pradesh), three from the east region (Bihar, West Bengal and Odisha), two each from the west (Gujarat and Maharashtra) and south region (Andhra Pradesh and Tamil Nadu). In addition to geographical representation, these states were chosen to represent the variation in net state domestic product (NSDP), health indicators and health workforce density across India. `The multi-stage sampling strategy also aimed to capture differences in cost associated with specialties and type of providers. The procedure followed for selection of each of the public (district and tertiary facilities) and private hospitals are explained in detail in the protocol paper [26].

A mixed methodology consisting of both bottom-up and top-down costing approaches were used for data collection, and standard analytical principles were applied [27, 28] The lack of disaggregated data on resource use and electronic health records in the Indian healthcare system led to the use of mixed costing approach. The unit cost of outpatient consultations, inpatient bed days and intensive care bed days were estimated using the top-down approach. The cost of an individual surgery was estimated using a mixed micro-costing approach. Under this, the data on the use of resources like equipment, drugs, and consumables for each surgery was captured using a bottom-up approach and the cost of human resources, infrastructure, furniture, and overheads was estimated using the top-down methods. Combining both the approaches provides with a sufficient degree of disaggregation of the estimated cost into its specific input resources, necessary for the purpose of setting reimbursement rates and HTA. The data collection was undertaken for the reference period of April 2017 to March 2018 across all the sampled hospitals. The details on the data collection methodology and data analysis plan of the CHSI study and a process evaluation of the quality and challenges faced during the data collection have also been published elsewhere [12, 26, 29].

### Analytical approach

We estimated speciality specific unit cost of services within each selected hospital. These unit costs included the cost-of-service delivery in four basic cost centres of outpatient department (OP), inpatient department (IP), intensive care unit (ICU) and operation theatre (OT) within a speciality. The cost-of-service delivery under each of the cost centres were computed following a standard classification of fixed and variable costs. Cost of input resources that are not dependent upon on the output produced, i.e., salaries of human resources, annualized cost of capital space, equipment (excluding the maintenance cost) and furniture were classified under the category of fixed costs. Further, the costs, which vary with the increase or decrease in the volume of output, e.g. drugs, consumables, utility, stationery, maintenance other supplies and overheads such as electricity, water, maintenance, etc., were classified as variable costs.

The unit costs of service delivery for each centre were computed based on the actual resource consumption and service utilization (i.e., current levels of capacity utilization) of the health facilities. However, as service utilisation (e.g., outpatient consultation, number of inpatient admissions both in inpatient wards and ICU) relative to the resources available (i.e., capacity utilisation) varies across similar services, specialities and facilities, unit costs were standardised to enable comparison. As bed occupancy rate is a standard indicator reflective of hospital service load, it was used to adjust for differences in capacity utilization for each of OP, IP, ICU and OT specific standardized unit costs [10, 30, 31]. Standardised unit costs were calculated using the service utilisation figures (the denominator in the unit cost) in line with bed occupancy rates of 80% and 100% of full capacity for each speciality. Bed occupancy rates were calculated based on actual data on the number of beds, average length of stay and patients admitted during the particular year. Under the standardization process, the cost incurred for variable resources such as drugs, consumables, utility, overheads, etc. were adjusted for the change in capacity utilisation while keeping cost of fixed assets in the form of space, equipment, furniture, and human resources constant. All costs were analysed in Indian Rupees, 2020 prices and converted to USD for presentation (1 USD = ₹ 76.21) [32].

A total of 327 specialties were included, with 48, 79 and 200 specialties covered in tertiary, private and district hospitals respectively (Table 1). Further, from these specialties, cost data collected from a total of 408 OP units, 327 IP units, 45 ICU units and 219 OT units were included in the analysis (Supplementary tables S1 – 3). Distribution of the cost centres by the type of provider, location of the facility (by the tier of the facility) and by specific facilities is presented in Table 1 and supplementary tables S1 –3.

**Table 1:** Profile of the sampled specialities at each facility by type of facility and tier city

	Number of departments/ specialities	Number of beds		ALOS		Bed occupancy	
		Median	IQ range	Median	IQ range	Median	IQ range
**Inpatient Cost Centre**							
Overall	327	22.0	(10.0-45.0)	3.9	(2.8-5.1)	0.7	(0.3-1.4)
** By type of facility**		*p* < 0.05		*p* < 0.05		*p* < 0.05	
District	200	27.0	(13.8-48.0)	4.3	(3.3-5.2)	0.8	(0.4-1.6)
Private	79	6.0	(3.0-11.0)	2.5	(2.0-3.0)	0.5	(0.3-0.9)
Tertiary	48	52.0	(32.3-18.3)	5.8	(4.0-7.0)	0.7	(0.5-1.2)
** By tier city**		*p* < 0.05		*p* < 0.05		*p* < 0.05	
Tier1	25	5.0	(4.3-5.5)	42.0	(30.0-63.0)	0.5	(0.3-0.7)
Tier2	81	3.0	(2.0-4.7)	11.0	(5.0-30.0)	0.5	(0.3-1.1)
Tier3	221	4.0	(3.0-5.2)	24.0	(10.0-45.0)	0.8	(0.4-1.6)
**ICU Cost Centre**							
Overall	45	14.0	(10.0-24.0)	3.5	(2.0-5.0)	0.6	(0.3-1.5)
** By type of facility**		*p* < 0.05		*p* < 0.05		*p* < 0.05	
District	19	14.0	(10.0-22.0)	4.6	(4.1-5.7)	0.7	(0.4-1.6)
Private	10	10.5	(9.3-14.0)	3.0	(2.9-3.0)	0.2	(0.1-0.4)
Tertiary	16	18.0	(12.8-25.3)	2.0	(2.0-3.3)	0.8	(0.4-1.6)
** By tier city**		ns		*p* < 0.05		*p* < 0.05	
Tier1	9	13.0	(12.0-24.0)	2.0	(2.0-2.0)	0.7	(0.4-1.5)
Tier2	11	13.0	(9.5-17.0)	3.0	(2.0-4.0)	0.6	(0.2-2.0)
Tier3	25	18.0	(10.0-29.0)	4.5	(3.0-5.7)	0.6	(0.3-1.5)

Note: *p* values are based on the Kruskal–Wallis test for difference between groups

A descriptive-analytical approach was used to present and summarise the cost data and to compare the influence of provider type on the unit costs at the specialty level for each cost centre. The role of capacity utilisation (bed occupancy) in driving the differences in unit costs across provider types was explored by comparing provider capacity utilisation unadjusted and adjusted costs. Next, the impact of the average length of stay (ALOS) on unit cost was examined by comparing the adjusted and unadjusted costs per admission and costs per bed day across the provider types. Finally, the impact of geography and price were explored by comparing the capacity utilisation adjusted costs per outpatient visit and cost per bed day across city tiers. The analysis presents the median unit costs and tests for differences using the Kruskal–Wallis test for small samples [33]. Unit costs also vary with scale of activity, as a result of economies of scale, in a non-linear fashion, to form a classic “u-shaped” average cost curve [34]. Where average costs are minimised, relative to the scale of activity, services are said to be scale efficient [35]. Scale efficiency was explored by testing for the likelihood of a non-linear relationship between scale and unit cost using Pearson’s rank correlation. In addition, scatter plots with LOWESS smoothing were generated to allow the visual assessment of the relationship. Lowess smoothing is a process built into statistical software that creates a line through the central tendency of the relationship between two variables [36]. Due to the need for large samples and to ensure comparability of service provision, the scale analysis was carried out for district hospitals only (*n* = 278). The analysis was also restricted to the inpatient and outpatient cost centres as the ICU sample was relatively small and small scale, while for the OT cost centre variable costs (costs that vary directly with the level of output/scale) are a significant proportion of costs so that economies of scale are unlikely. Scale variables used were number of visits, number of admissions, number of beds and bed occupancy. Analysis was carried out using RStudio [36].

### Ethics and consent

The hospital cost data used for analysis was collected under the Costing Health Services in India study for the Department of Health Research, New Delhi. Ethics approval was approved by the Institutional Ethics Committee (IEC) vide letter no. PGI/IEC/2018/00125A and Institutional Collaborative Committee (ICC) vide letter no. 79/30-Edu-13/111273 of Postgraduate Institute of Medical Education and Research, Chandigarh, India. No individual (human) level data was used in the analysis. All methods were carried out in accordance with relevant guidelines and regulations.

### Patient and public involvement

Neither patients or public were involved in the design, conduct, reporting or dissemination plans of our research.

## Results

### Profile of specialties across sampled hospitals

The median number of beds was 52, 6 and 27 beds per speciality in tertiary, private and district hospitals, respectively (Table 1). The average IPD length of stay (ALOS) was higher for tertiary care facilities (5.8) compared to both district (4.3) and private hospitals (2.5). The bed occupancy rate in IPD was lower in private hospitals (50%) compared to both district (80%) and tertiary level facilities (70%) (Table 1). Similarly, the bed occupancy rate in ICU was lower in private hospitals (20%) compared to both district (70%) and tertiary level facilities (80%). The average number of annual OPD consultations (per speciality) was highest in public tertiary (48,866), followed by public district (17,250) and private hospitals (1142) (Supplementary material; Table S3). Similarly, the average annual number of surgical procedures (per speciality) conducted in OT were highest in public tertiary (2389), followed by district (461) and private facilities (269) (Supplementary material; Table S3). Profile of the facilities across hospitals by the tier of the city is presented in Table 1 and supplementary material; tables S1 – 3.

### Cost centre unit costs by type of provider

**Fig. 1:**
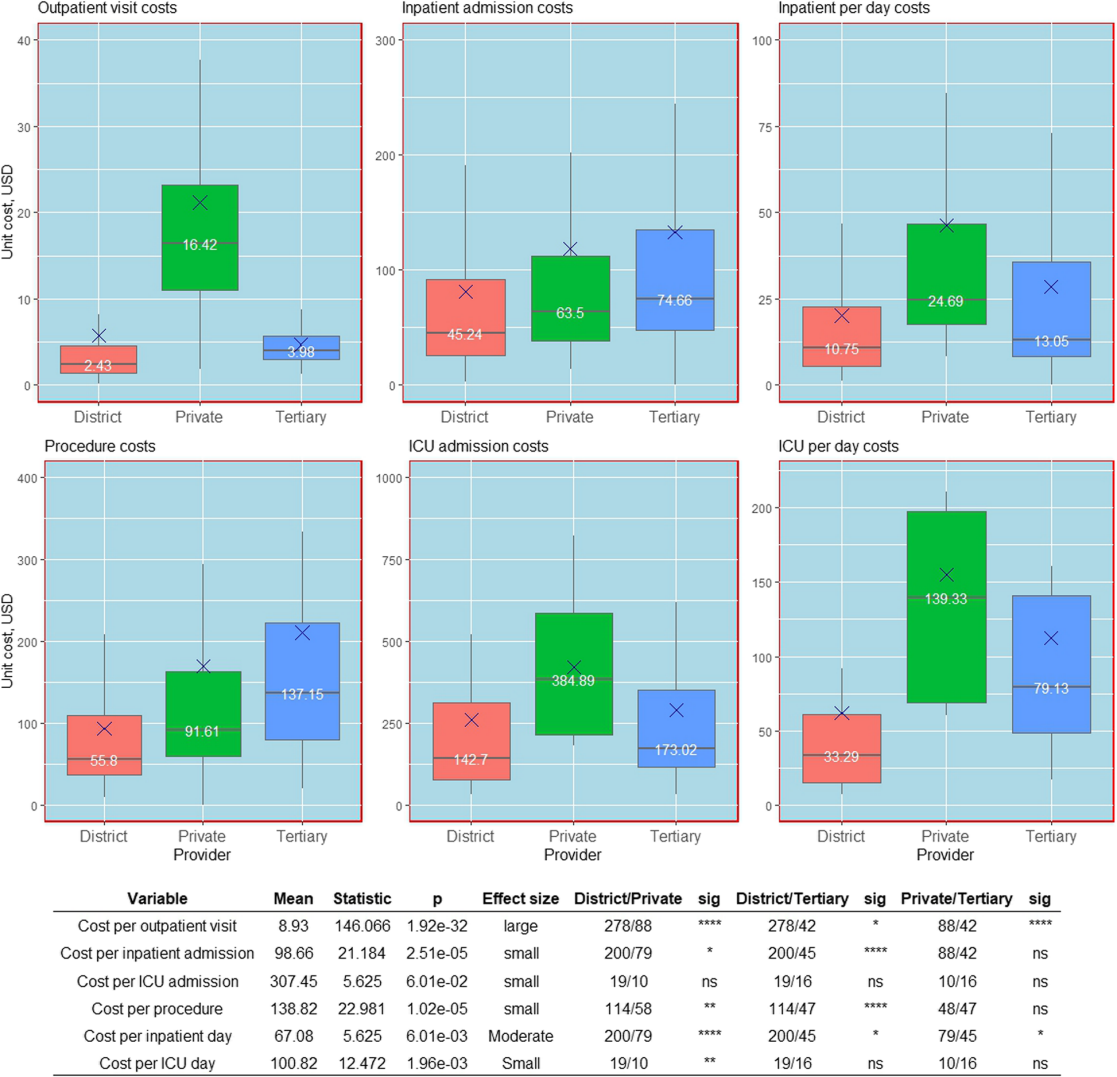
Unadjusted unit costs (USD) for the outpatient, inpatient, operating theatre and intensive care unit cost centres by type of provider. Kruskal–Wallis test was used to test for the significant differences between groups and the effect size—*p* values are based on the Kruskal–Wallis test for difference between groups. The size of the effect is derived from the Eta squared statistic where eta2 [H] = (H—k + 1)/(n—k); H is the value obtained in the Kruskal–Wallis test; k is the number of groups; n is the total number of observations (0.01- < 0.06 (small effect), 0.06—< 0.14 (moderate effect) and >  = 0.14 (large effect))

**Fig. 2:**
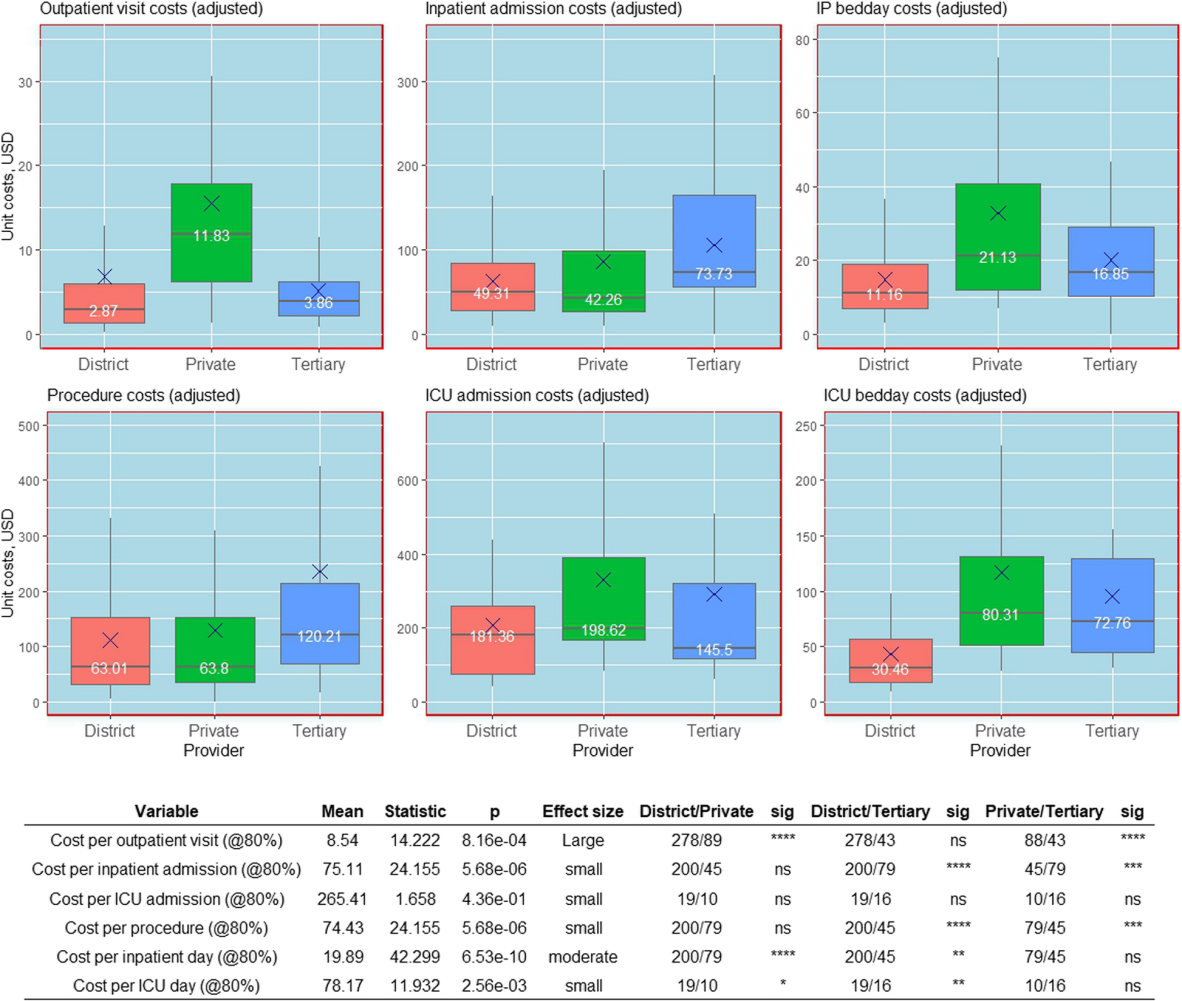
Adjusted unit costs (USD) for the outpatient, inpatient, operating theatre and intensive care unit cost centres by type of provider. Unit costs are adjusted to account for differences in capacity utilisation as measured by the bed occupancy rate, by recalculating the unit cost with bed occupancy rates of 80%. Kruskal–Wallis test was used to test for the significant differences between groups and the effect size—*p* values are based on the Kruskal–Wallis test for difference between groups. The size of the effect is derived from the Eta squared statistic where eta2 [H] = (H—k + 1)/(n—k); H is the value obtained in the Kruskal–Wallis test; k is the number of groups; n is the total number of observations (0.01- < 0.06 (small effect), 0.06—< 0.14 (moderate effect) and >  = 0.14 (large effect))

Figures 1 and 2 show the median values and variation in unadjusted and adjusted (at 80% capacity) unit costs of service delivery in OPD, IPD, ICU and OT cost centres within a speciality by the type of hospital, respectively. The median unadjusted cost per outpatient visit was highest in private hospitals (₹ 1251, 16 USD) followed by tertiary care facilities (₹ 304, 4 USD) and district hospitals (₹ 185, 2.43 USD). This difference in unadjusted OPD costs was statistically significant across all the provider types. After adjusting for capacity utilisation, the difference in the outpatient visit cost between the district and tertiary care facilities became statistically insignificant (*p* = 0.3831).

The unadjusted cost per admission in IPD was higher for tertiary facilities (₹ 5690, 75 USD) followed by private facilities (₹ 4839, 63 USD) and district hospitals (₹ 3447, 45 USD), with an insignificant difference between tertiary and private hospitals. However, following adjustment for capacity utilisation, tertiary level costs (₹ 5619, 74 USD) became significantly higher than for both the district and private hospitals. There was no significant difference in the cost per admission in ICU among the type of providers for both unstandardized and standardized scenarios, with an overall unadjusted mean value of ₹ 23,431 (307 USD).

In contrast to overall admission costs, the unadjusted cost per IPD bed day was highest in private facilities (₹ 1882, 25 USD) followed by tertiary (₹ 995, 13 USD) and district hospitals (₹ 819, 11 USD) and the adjusted costs followed a similar pattern. For the unadjusted costs, the difference was significant across all provider types. However, the adjusted unit cost did not differ significantly between private and tertiary care facilities. Likewise, the unadjusted cost per ICU bed-day was highest in private facilities (₹ 10,619, 139 USD) followed by tertiary (₹ 6031, 79 USD) and district hospitals (₹ 2537, 33 USD). But the difference was only statistically significant between the district and private hospitals. After adjusting, the difference in cost between district and tertiary facilities also became significant.

The unadjusted cost per procedure was significantly higher in tertiary facilities (₹ 10,452, 137 USD) compared to district hospitals (₹ 4253, 56 USD) but not private (₹ 6982, 92 USD) hospitals. Further, the difference in cost between the district and private was also significant. However, after adjusting for capacity, the trend was reversed. The difference between private and tertiary facilities became significant and the difference in cost between the district and private facilities turned insignificant.

### Geographical variation in the cost of healthcare

**Fig. 3:**
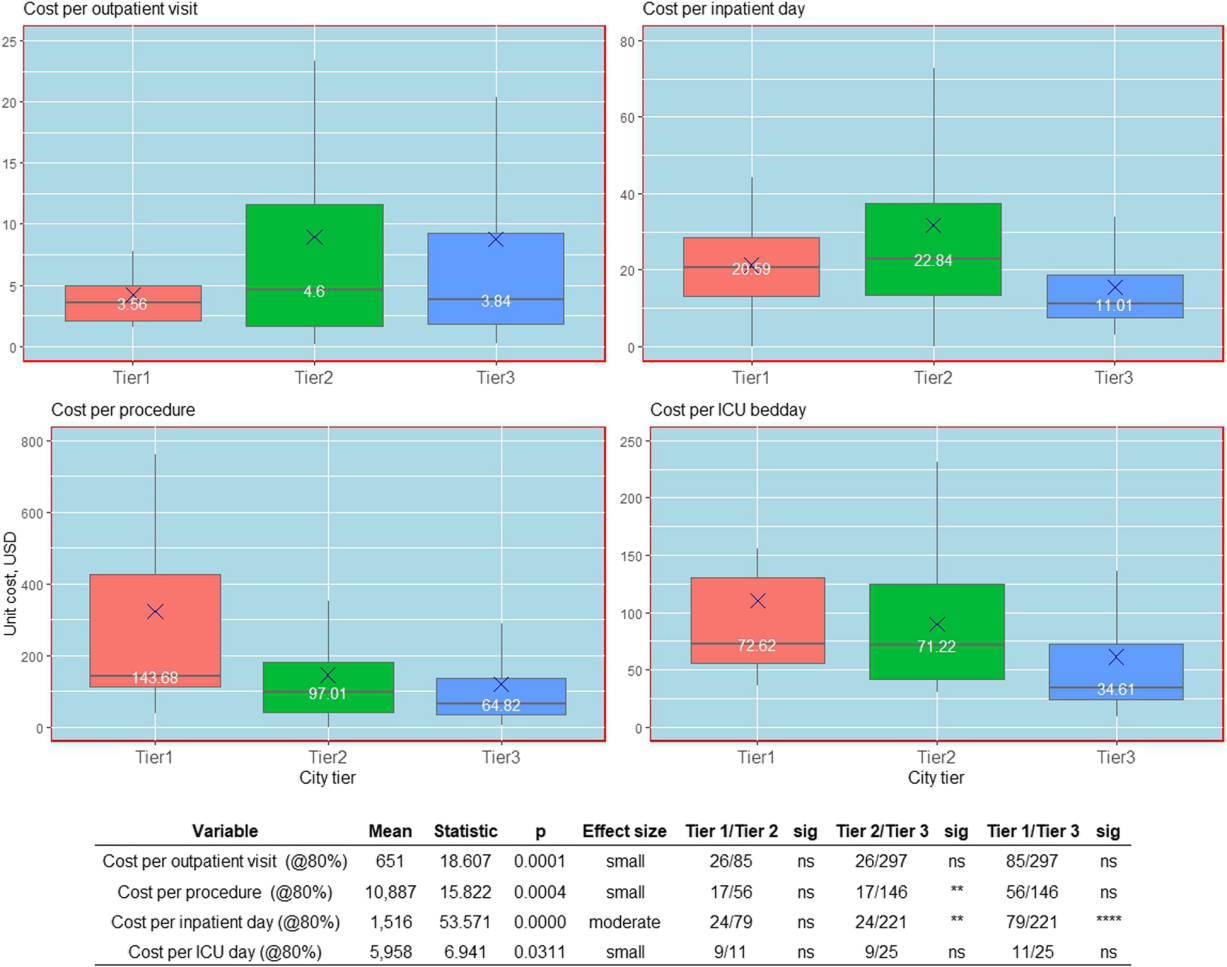
Adjusted unit costs (USD) for the outpatient, inpatient, operating theatre and intensive care unit cost centres by tier city. Unit costs are adjusted to account for differences in capacity utilisation as measured by the bed occupancy rate, by recalculating the unit cost with bed occupancy rates of 80%. The Kruskal–Wallis test was used to test for the significant differences between groups and the effect size—*p* values are based on the Kruskal–Wallis test for difference between groups. The size of the effect is derived from the Eta squared statistic where eta2 [H] = (H—k + 1)/(n—k); H is the value obtained in the Kruskal–Wallis test; k is the number of groups; n is the total number of observations (0.01- < 0.06 (small effect), 0.06—< 0.14 (moderate effect) and >  = 0.14 (large effect))

Figure 3 presents the standardized cost centre unit costs by the provider's location according to the tier of the city. There was no statistically significant difference in cost per outpatient consultation within a speciality for hospitals located in tier 2 (₹ 350, 4.6 USD), tier 3 (₹ 293, 3.8 USD) and tier 1 (₹ 271, 3.6 USD) city. In the case of procedures, tier 1 city facilities had the highest costs (₹ 10,950, 144 USD) and tier 3 facilities had the lowest (₹ 4940, 65 USD), but were only significantly different between tier 2 and tier 3. The cost per bed day in IPD was also highest for hospitals located in tier 2 city (₹ 1741, 23 USD), followed by hospitals in tier 1 (₹ 1569, 21 USD) and tier 3 (₹ 839, 11 USD) city. This difference in the cost was significant for hospitals in tier 3 cities compared to both tier 2 and tier 1 cities, with no significant difference for hospitals between tier 2 and tier 3 cities. The ICU bed day cost was highest for hospitals in tier 1 (₹ 5534, 73 USD) city followed by tier 2 (₹ 5427, 71 USD) and tier 3 (₹ 2638, 35 USD) cities, although this difference in costs was insignificant.

### Impact of scale on cost

**Fig. 4:**
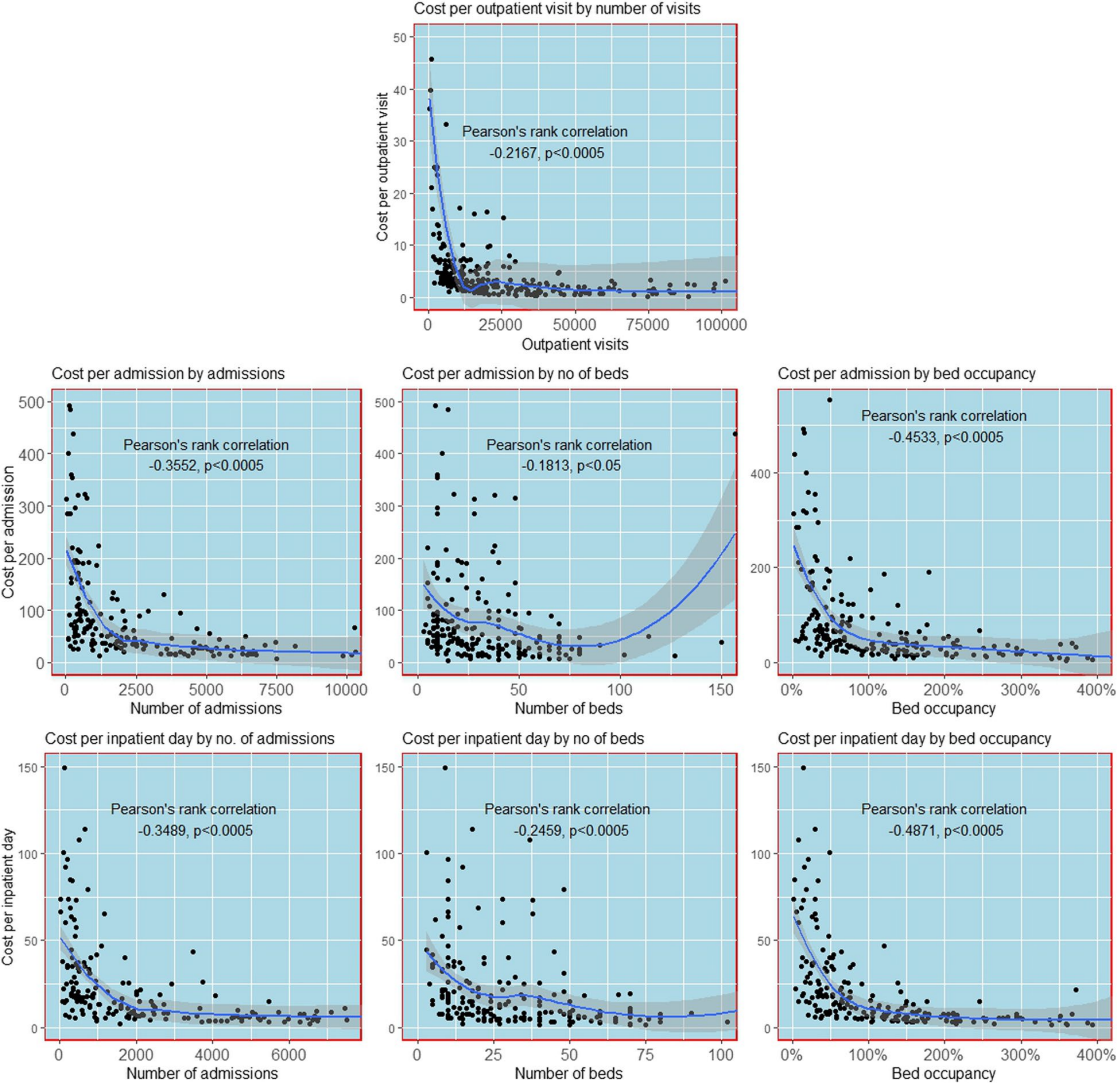
The impact of scale on per bed-day and admission costs (USD; excluding outliers). Regression lines generated using LOWESS smoothing in Rstudio

The cost per bed-day in IPD within a speciality was inversely correlated with an increase in the number of admissions (*r* = -0.34; *p* < 0.0005) and bed occupancy rate (*r* = -0.487; *p* < 0.0005), as shown in Fig. 4. The cost per outpatient visit compared with the number of outpatient visits and per bed-day IPD cost compared with the number of beds showed weak negative correlations (*r* = -0.25; *p* < 0.0005 and-0.2167; *p* < 0.0005, respectively). In addition, cost per admission in IPD also showed a moderate and inverse correlation with the number of admissions (*r* = -0.35; *p* < 0.0005) and bed occupancy rates (*r* = -0.453; *p* < 0.0005). However, there was a weak correlation of IPD admission cost (*r *= -0.18; *p* < 0.05) with the increase in the number of beds within a speciality. The LOWESS smoothing confirms that the relationships between unit cost and scale are not linear.

## Discussion

These unique cost data from across 11 states and 54 health care providers of different types in India provide an important contribution to understanding the heterogeneity in health care costs in India, being the largest multisite facility costing study including both public and private providers. As publicly funded insurance programmes expand at the national and state level, the importance of this type of cost information to inform health benefit package design, pricing of health services and HTA and to design appropriate provider incentives has become increasingly important. The descriptive analysis provides cost data for further empirical analysis including economic evaluation, price setting and drivers of technical efficiency.

The results confirm the heterogeneity in costs for each of the cost centres across different provider types. The private sector hospitals often set high prices for the provision of care. However, our study findings show that cost per admission and procedure, after adjustment for capacity utilization, are higher in public tertiary hospitals. The cost of ICU care is similar across public tertiary and private hospitals. The descriptive analytics also help to explain how the scale (e.g. number of visits, number of admissions) and use of capacity (bed occupancy) influence unit costs and need to be accounted for when using cost information for HTA and price setting to avoid incentivising inefficiencies [37]. Bed occupancy rates and length of stay are strikingly low in the private sector compared to public hospitals and provide important evidence of differing approaches to patient management and efficiency. Lower bed occupancy rates in the private sector appear to explain higher admission costs and suggest excess capacity in the private sector. On the other hand, lower lengths of stay in the private sector result in bed day costs that are comparable across the private sector and tertiary facilities. However, without understanding any possible variations in case mix, this does not necessarily mean levels of technical efficiency are comparable. As public tertiary facilities are a “last resort” point of care, it is possible that they see more complex cases than the sampled private facilities, none of which are tertiary providers. Our study findings highlight the need for better cost accounting systems in the private sector to determine the actual cost, and to understand the basis of pricing decisions.

Using cost information as generated by the CHSI study to inform the price is a way to help ensure the correct price signals and to do so transparently so that purchasers avoid incentivising less than optimal efficiency. Further, purchasers also need to compensate for price differentials that are beyond the control of the individual provider. In India, cities are classified into 3 tiers, by the National Pay Commission, [38] according to the cost of living, to set the level of government staff allowances. The comparative analysis of costs in the different tier cities found significant differences in adjusted bed day costs when comparing tier 3 cities with the tier 1 and tier 2 cities, and adjusted procedure costs when comparing tier 2 and tier 3 cities. As health benefit package prices for AB-PMJAY are largely made of inpatient and procedure costs, [12] this supports an argument for adjusting rates for tier 1 and tier 2 city facilities above the rates of tier 3 city sites. The lack of difference between tier 1 and tier 2 is not intuitive so it is possible the lack of significance results from a relatively small sample of tier 1 hospitals (25 specialities from 5 hospitals). Further data is likely needed to confirm these findings as well as to inform the level of the price weight.

The analysis has looked at the scale of activity in the inpatient cost centre as represented by the number of admissions, number of beds and bed occupancy. The combined results of Pearson’s rank and LOWESS smoothing, confirms that the inpatient care in India is no exception to the rule that scale of activity is known to be a driver of hospital costs and technical efficiency [34, 39]. These scale differences need to be accounted for in economic evaluations and can also inform central guidance on hospital size at each level of the system or potentially geographical areas using a statistical cost function or frontier approach [40].

### Limitations

Descriptive cost analysis provides an overview of the current patterns of actual resource use. However, as with all cost analyses, these data do not necessarily reflect the provision of efficient, good quality services and it is not possible to determine the role of quality in driving any differences in cost. In addition, due to the heterogeneity of facility costs, there is no clear-cut guidance on the sample size calculation for health facility studies. As a result, the sampling approach was designed to ensure that the estimates were as generalisable as possible but cannot be defined as nationally representative as in a population-based study. The findings need to be understood within this context.

Due to the sample size limitations, our analysis does not explore the differences in cost at the specialty or state levels. However, for those specialties with large enough samples, a comparison of unadjusted and adjusted inpatient day costs is presented in the supplementary material (table S4) which shows results consistent with the overall findings. In addition, the costs by specialty and by state are provided in the supplementary material to show the range of costs (tables S1-3 and S5). One further limitation of the CHSI data is that very large (more than 250 beds) private hospitals located in metro cities were not included. The private sector in India is mixed and does include tertiary level facilities with multiple specialties. However, as the focus of PM-JAY is to ensure that there is sufficient empanelment of hospitals and coverage in tier 2 and 3 cities where private providers tend to be smaller in size, our study findings are relevant to policy discussions.

### Policy implications and implications for future research

In setting prices for the HBPs under AB-PMJAY, there has been significant discourse about the inadequacy of the rates and some debate on the use of cost information from public sector facilities to help inform the process. The findings presented here show that after adjustment for capacity utilization, though the private hospitals still had higher OPD unit costs than the public tertiary hospitals, the per bed day costs were similar between these facilities, and tertiary hospitals had a higher cost per procedure. In addition, the cost per bed day (both in IPD and ICU) and cost per procedure were significantly higher in tertiary facilities but with a similar cost of outpatient consultation than in district hospitals. As a result, the estimates of cost derived from the analysis of data from public sector hospitals should be sufficient to cover the provider payment rates of the HBPs in any facility. This is more so as preliminary estimates from these data show that the procedure cost constitutes more than 66% of the total cost of surgical HBPs based on cost data from public tertiary care facilities. The analysis also confirms that geographical location does explain some of the heterogeneity and the justification of the price weights currently used to account for these differences in AB-PMJAY. Further analysis using the CHSI data is currently underway using regression methods to inform the size of these weights. [41].

From the perspective of economic evaluations and HTA more broadly, evidence from the sensitivity analyses shows that variation in costs can significantly shift results by directly influencing the value of the incremental cost-effectiveness ratio [3, 42, 43]. A review of 13 HTAIn commissioned studies, found that the cost data used in the majority of these studies, whether drawn from primary data collection or secondary data, was based on the analysis of cost information collected from a single or two hospitals [42]. The appraisal of HTA studies must consider the costing methodology and sampling followed to account for the large extent of heterogeneity in health care costs [44]. With the production of the CHSI data, a national effort to add these to an online platform for sharing these nation-specific cost data for use in HTA, price-setting and other planning activities is ongoing [45, 46].

## Conclusion

The reliance on cost information from single sites or small samples ignores the issue of heterogeneity driven by both demand and supply-side factors. The CHSI cost data set provides a unique insight into cost variability across different types of providers in India to assist in healthcare decision making from budgeting to economic evaluation and price-setting.

## Supplementary Information

**Additional File 1: Table S1:** Profile of sampled specialties (Inpatient cost centre)- all costs in INR. **Table S2** Profile of sampled specialties (ICU cost centre) -all costs in INR. **Table S3 **Profile of sampled specialties (OP and OT cost centres) – all costs in INR. **Table S4 **Comparison of unadjusted and adjusted cost per bed day (Inpatient cost centre) for selected specialties (INR).** Table S5 **Unadjusted unit costs by state and type of provider (INR).

## Data Availability

All the cost information generated by the study is included in the paper and supplementary material. The code availability is not applicable.
