# Supplementary material for: Cost of hospital services in India: a multi-site study to inform provider payment rates and Health Technology Assessment
Source: BMC Health Serv Res. 2022 Nov 14;22:1343. doi: 10.1186/s12913-022-08707-7 (PMC9664599; doi:10.1186/s12913-022-08707-7)
Supplement: Supplementary file 1 — Additional File 1: Table S1: Profile of sampled specialties (Inpatient cost centre)- all costs in INR. Table S2 Profile of sampled specialties (ICU cost centre) -all costs in INR. Table S3 Profile of sampled specialties (OP and OT cost centres) – all costs in INR. Table S4 Comparison of unadjusted and adjusted cost per bed day (Inpatient cost centre) for selected specialties (INR). Table S5 Unadjusted unit costs by state and type of provider (INR). [file 12913_2022_8707_MOESM1_ESM.docx]

# Efficiency and Cost of Hospital Services in India: A Multi-Site Study to Inform Provider Payment Rates and Health Technology Assessment

Supplementary material

**Supplementary material: Table S1 Profile of sampled specialties (Inpatient cost centre)- all costs in INR**

|  | **N** | **Number of beds** | | | **Average length of stay** | | | **Bed occupancy** | | | **Cost per admission (unadjusted)** | | |
| --- | --- | --- | --- | --- | --- | --- | --- | --- | --- | --- | --- | --- | --- |
| **Category** |  | **Median** | **IQ range** | | **Median** | **IQ range** | | **Median** | **IQ range** | | **Median** | **IQ range** | |
| Overall | 327 | 22 | (10 | - 45) | 3.9 | (2.8 | - 5.1) | 0.7 | (0.3 | - 1.4) | 3,926 | (2,319 | - 8,057) |
| **Provider type** | | | | | | | | | | | | | |
| District | 200 | 27 | (14 | - 48) | 4.3 | (3.3 | - 5.2) | 0.8 | (0.4 | - 1.6) | 3,447 | (1,984 | - 7,012) |
| Private | 79 | 6 | (3 | - 11) | 2.5 | (2.0 | - 3.0) | 0.5 | (0.3 | - 0.9) | 4,839 | (2,922 | - 8,563) |
| Tertiary | 48 | 52 | (32 | - 118) | 5.8 | (4.0 | - 7.0) | 0.7 | (0.5 | - 1.2) | 5,690 | (3,609 | - 10,290) |
| **City classification** | | | | | | | | | | | | | |
| Tier1 | 25 | 42 | (30 | - 63) | 5.0 | (4.3 | - 5.5) | 0.5 | (0.3 | - 0.7) | 7,218 | (4,913 | - 18,245) |
| Tier2 | 81 | 11 | (5 | - 30) | 3.0 | (2.0 | - 4.7) | 0.5 | (0.3 | - 1.1) | 7,249 | (3,943 | - 11,648) |
| Tier3 | 221 | 24 | (10 | - 45) | 4.0 | (3.0 | - 5.2) | 0.8 | (0.4 | - 1.6) | 3,180 | (2,016 | - 5,976) |
| **Specialty** | | | | | | | | | | | | | |
| OBG | 55 | 48 | (16 | - 68) | 4.2 | (3.0 | - 5.1) | 1.0 | (0.6 | - 1.8) | 2,670 | (1,661 | - 6,463) |
| Gen | 49 | 28 | (9 | - 50) | 4.0 | (2.9 | - 5.3) | 0.7 | (0.4 | - 1.1) | 3,857 | (2,863 | - 7,482) |
| Med | 39 | 36 | (16 | - 50) | 3.5 | (3.0 | - 4.4) | 1.0 | (0.6 | - 1.6) | 2,961 | (1,779 | - 6,074) |
| Ortho | 39 | 22 | (10 | - 40) | 5.3 | (3.0 | - 6.7) | 0.6 | (0.3 | - 1.3) | 4,967 | (2,968 | - 7,588) |
| Ophth | 29 | 22 | (10 | - 27) | 2.8 | (1.7 | - 4.3) | 0.4 | (0.3 | - 0.6) | 3,905 | (2,959 | - 5,231) |
| Paed | 29 | 29 | (20 | - 40) | 3.8 | (3.4 | - 4.6) | 1.3 | (0.6 | - 1.5) | 2,877 | (1,206 | - 5,940) |
| ENT | 24 | 10 | (8 | - 22) | 3.8 | (2.8 | - 4.3) | 0.4 | (0.3 | - 0.8) | 5,150 | (4,212 | - 8,195) |
| Chest | 17 | 14 | (10 | - 24) | 5.1 | (3.8 | - 5.5) | 0.8 | (0.2 | - 1.6) | 9,346 | (2,788 | - 16,426) |
| Uro | 10 | 19 | (1 | - 40) | 4.0 | (2.6 | - 5.0) | 0.5 | (0.3 | - 1.0) | 8,493 | (7,243 | - 13,966) |
| Card | 7 | 30 | (5 | - 55) | 3.0 | (2.7 | - 4.0) | 0.6 | (0.5 | - 1.4) | 3,115 | (2,726 | - 6,284) |
| Emergency | 7 | 13 | (10 | - 21) | 1.8 | (1.5 | - 2.3) | 2.8 | (1.4 | - 3.7) | 2,449 | (1,105 | - 2,831) |
| Burn | 5 | 10 | (5 | - 10) | 6.4 | (4.8 | - 6.5) | 0.3 | (0.2 | - 0.8) | 11,677 | (7,002 | - 16,743) |
| Dental | 4 | 3 |  |  | 1.0 |  |  | 0.3 |  |  | 4,879 |  |  |
| Nephrology | 4 | 8 |  |  | 3.5 |  |  | 0.6 |  |  | 3,256 |  |  |
| CTVS | 3 | 42 |  |  | 8.0 |  |  | 0.3 |  |  | 42,179 |  |  |
| Geriatric | 3 | 10 |  |  | 3.0 |  |  | 0.1 |  |  | 3,420 |  |  |
| Gastrology | 1 | 6 |  |  | 5.0 |  |  | 0.8 |  |  | 3,366 |  |  |
| Neurology | 1 | 2 |  |  | 1.5 |  |  | 0.3 |  |  | 7,275 |  |  |
| Psychiatry | 1 | 10 |  |  | 5.1 |  |  | 0.7 |  |  | 6,305 |  |  |

**Supplementary material: Table S2 Profile of sampled specialties (ICU cost centre) -all costs in INR**

|  | **N** | **Number of beds** | | | **Average length of stay** | | | **Bed occupancy** | | | **Cost per ICU admission (unadjusted)** | | |
| --- | --- | --- | --- | --- | --- | --- | --- | --- | --- | --- | --- | --- | --- |
| **Category** |  | **Median** | **IQ range** | | **Median** | **IQ range** | | **Median** | **IQ range** | | **Median** | **IQ range** | |
| Overall | 45 | 14 | (10 | - 24) | 3.5 | (2.0 | - 5.0) | 0.6 | (0.3 | - 1.5) | 15,535 | (9,565 | - 30,973) |
| **Provider type** | | | | | | | | | | | | | |
| District | 19 | 14 | (10 | - 22) | 4.6 | (4.1 | - 5.7) | 0.7 | (0.4 | - 1.6) | 10,875 | (6,030 | - 23,947) |
| Private | 10 | 11 | (9 | - 14) | 3.0 | (2.9 | - 3.0) | 0.2 | (0.1 | - 0.4) | 29,333 | (16,378 | - 44,806) |
| Tertiary | 16 | 18 | (13 | - 25) | 2.0 | (2.0 | - 3.3) | 0.8 | (0.4 | - 1.6) | 13,186 | (8,999 | - 26,734) |
| **City classification** | | | | | | | | | | | | | |
| Tier1 | 9 | 13 | (12 | - 24) | 2.0 | (2.0 | - 2.0) | 0.7 | (0.4 | - 1.5) | 19,771 | (9,617 | - 24,448) |
| Tier2 | 11 | 13 | (10 | - 17) | 3.0 | (2.0 | - 4.0) | 0.6 | (0.2 | - 2.0) | 23,579 | (11,148 | - 38,081) |
| Tier3 | 25 | 18 | (10 | - 29) | 4.5 | (3.0 | - 5.7) | 0.6 | (0.3 | - 1.5) | 13,877 | (8,429 | - 30,973) |
| **Specialty** | | | | | | | | | | | | | |
| ICU | 27 | 12 | (10 | - 20) | 4.1 | (3.0 | - 5.4) | 0.6 | (0.2 | - 1.4) | 18,906 | (10,123 | - 35,348) |
| OBG | 5 | 18 | (16 | - 20) | 2.0 | (2.0 | - 3.0) | 0.7 | (0.6 | - 0.9) | 11,292 | (10,191 | - 12,106) |
| Card | 4 | 21 |  |  | 2.0 | (2.0 | - 2.5) | 1.9 |  |  | 6,342 |  |  |
| CTVS | 3 | 24 |  |  | 2.0 | (2.0 | - 2.0) | 0.1 |  |  | 24,448 |  |  |
| Gen | 2 | 15 |  |  | 4.0 | (3.0 | - 5.0) | 1.0 |  |  | 31,614 |  |  |
| Ortho | 2 | 10 |  |  | 4.8 | (3.9 | - 5.6) | 1.1 |  |  | 37,627 |  |  |
| Paed | 1 | 30 |  |  | 4.6 | (4.6 | - 4.6) | 2.0 |  |  | 2,562 |  |  |
| Uro | 1 | 19 |  |  | 2.0 | (2.0 | - 2.0) | 0.7 |  |  | 9,617 |  |  |

**Supplementary material: Table S3 Profile of sampled specialties (OP and OT cost centres) – all costs in INR**

|  | **Outpatient cost centre** | | | | | | | **Operating theatre cost centre** | | | | | | |
| --- | --- | --- | --- | --- | --- | --- | --- | --- | --- | --- | --- | --- | --- | --- |
|  |  | **Number of visits** | | | **Cost per visit** | | | **Number of procedures** | | | | **Cost per procedure** | | |
| **Category** | **N*** | **Median** | **IQrange** | | **Median** | **IQrange** | | **N** | **Median** | **IQrange** | | **Median** | **IQrange** | |
| Overall | 408 | 13,915 | (4,519 | - 35,364) | 258 | (141 | - 597) | 219 | 599 | (216 | - 1,783) | 6,211 | (3,543 | - 11,497) |
| **Provider type** | | | | | | | | | | | | | | |
| District | 278 | 17,250 | (8,331 | - 36,242) | 185 | (109 | - 349) | 114 | 461 | (195 | - 1,580) | 4,253 | (2,832 | - 8,379) |
| Private | 88 | 1,142 | (508 | - 2,685) | 1,251 | (838 | - 1,771) | 58 | 269 | (121 | - 554) | 6,982 | (4,602 | - 12,438) |
| Tertiary | 42 | 48,866 | (29,838 | - 101,783) | 304 | (223 | - 433) | 47 | 2,389 | (1,434 | - 4,454) | 10,452 | (6,098 | - 16,947) |
| **City classification** | | | | | | | | | | | | | | |
| Tier1 | 26 | 34,415 | (14,916 | - 57,436) | 317 | (221 | - 483) | 17 | 2,041 | (1,170 | - 3,702) | 15,198 | (8,971 | - 23,075) |
| Tier2 | 85 | 5,995 | (1,248 | - 36,920) | 463 | (191 | - 1,221) | 56 | 555 | (224 | - 2,493) | 9,542 | (4,870 | - 16,074) |
| Tier3 | 297 | 13,120 | (5,774 | - 32,181) | 229 | (131 | - 515) | 146 | 482 | (186 | - 1,449) | 4,867 | (2,974 | - 8,407) |
| **Specialty** | | | | | | | | | | | | | | |
| Gen | 49 | 12,984 | (2,380 | - 31,500) | 279 | (164 | - 948) | 45 | 629 | (321 | - 1,551) | 5,956 | (3,410 | - 12,620) |
| OBG | 48 | 12,598 | (3,828 | - 35,762) | 295 | (157 | - 577) | 49 | 1,803 | (735 | - 5,881) | 4,154 | (3,075 | - 7,609) |
| Ortho | 44 | 16,190 | (5,782 | - 35,879) | 207 | (136 | - 437) | 38 | 318 | (178 | - 983) | 7,383 | (4,970 | - 15,540) |
| Med | 41 | 27,554 | (5,995 | - 63,553) | 172 | (98 | - 606) |  |  |  |  |  |  |  |
| Ophth | 35 | 17,229 | (7,127 | - 30,619) | 208 | (152 | - 463) | 31 | 667 | (376 | - 1,211) | 4,233 | (2,913 | - 7,793) |
| ENT | 33 | 15,547 | (7,140 | - 27,650) | 182 | (96 | - 279) | 32 | 128 | (39 | - 362) | 7,559 | (3,616 | - 10,471) |
| Paed | 33 | 32,304 | (19,051 | - 58,473) | 159 | (95 | - 350) |  |  |  |  |  |  |  |
| Dental | 32 | 8,710 | (5,738 | - 13,369) | 252 | (167 | - 545) | 3 | 5 | (4 | - 53) | 9,398 | (6,076 | - 78,337) |
| Emergency | 24 | 15,762 | (4,120 | - 65,574) | 532 | (247 | - 1,226) |  |  |  |  |  |  |  |
| Chest | 18 | 6,688 | (3,055 | - 15,284) | 372 | (178 | - 555) |  |  |  |  |  |  |  |
| Psychiatry | 18 | 6,442 | (3,860 | - 8,421) | 359 | (261 | - 583) | 1 | 2 | (2 | - 2) | 8,386 | (8,386 | - 8,386) |
| Uro | 10 | 7,686 | (513 | - 35,165) | 456 | (303 | - 1,516) | 12 | 215 | (115 | - 1,451) | 11,795 | (6,619 | - 22,614) |
| Card | 9 | 40,320 | (4,320 | - 95,497) | 252 | (212 | - 1,075) | 5 | 2,597 | (1,967 | - 6,519) | 11,656 | (10,038 | - 16,481) |
| Geriatric | 4 | 15,564 |  |  | 240 |  |  |  |  |  |  |  |  |  |
| CTVS | 3 | 13,713 |  |  | 492 |  |  | 3 | 848 | (722 | - 2,356) | 104,988 | (60,093 | - 109,831) |
| Nephrology | 3 | 710 |  |  | 1,422 |  |  |  |  |  |  |  |  |  |
| Neurology | 2 | 1,170 |  |  | 5,540 |  |  |  |  |  |  |  |  |  |
| Gastrology | 1 | 4,320 |  |  | 1,196 |  |  |  |  |  |  |  |  |  |
| Rheumatology | 1 | 1,560 |  |  | 463 |  |  |  |  |  |  |  |  |  |

*Note: The number of OP cost centres is larger than the number of IP cost centres as there are somespecialties evaluated for which no inpatient cost centre was present for the specialty.

**Supplementary material: Table S4 Comparison of unadjusted and adjusted cost per bed day (Inpatient cost centre) for selected specialties (INR)**

|  | Specialty | Kruskal-Wallis test  for difference in distributions | | | Effect size | District / Private | | | District / Tertiary | | | Private/ Tertiary | | |
| --- | --- | --- | --- | --- | --- | --- | --- | --- | --- | --- | --- | --- | --- | --- |
|  |  | Mean | Statistic | p |  | n | n | Signif. | n | n | Signif. | n | n | Signif. |
| Cost per bed day  (Inpatient cost centre) | Gen | 1,840.16 | 11.1839 | 0.00373 | large | 27 | 16 | ** | 27 | 6 | ns | 16 | 6 | ns |
|  | Med | 1,476.76 | 8.9177 | 0.00282 | large | 25 | 14 | ** |  |  |  |  |  |  |
|  | OBG | 1,457.21 | 14.7491 | 0.000627 | large | 29 | 14 | *** | 29 | 9 | ns | 14 | 9 | ns |
|  | Ortho | 1,739.01 | 15.4796 | 0.000435 | large | 21 | 12 | *** | 21 | 6 | ns | 12 | 6 | ns |
| Adjusted cost per bed day  (Inpatient cost centre) | Gen | 1,489.73 | 9.1611 | 0.0102 | large | 27 | 16 | * | 27 | 6 | ns | 16 | 6 | ns |
|  | Med | 1,341.27 | 4.4434 | 0.035 | moderate | 25 | 14 | * |  |  |  |  |  |  |
|  | OBG | 1,239.41 | 11.6402 | 0.00297 | large | 29 | 14 | ** | 29 | 9 | ns | 14 | 9 | ns |
|  | Ortho | 1,319.10 | 11.0649 | 0.00396 | large | 21 | 12 | ** | 21 | 6 | ns | 12 | 6 | ns |

|  | Specialty | Kruskal-Wallis test  for difference in distributions | | | Effect size | Tier 1/ Tier 2 | | | Tier 1/ Tier 3 | | | Tier 2/ Tier 3 | | |
| --- | --- | --- | --- | --- | --- | --- | --- | --- | --- | --- | --- | --- | --- | --- |
|  |  | Mean | Statistic | p |  | n | n | Signif. | n | n | Signif. | n | n | Signif. |
| Cost per bed day  (Inpatient cost centre) | Gen | 1,840.16 | 9.4511 | 0.00887 | large | 3 | 13 | ns | 3 | 33 | ns | 13 | 33 | ** |
|  | Med | 1,476.76 | 10.9135 | 0.00427 | large | 1 | 9 | ns | 1 | 29 | ns | 9 | 29 | ** |
|  | OBG | 1,457.21 | 2.4537 | 0.293 | small | 4 | 17 | ns | 4 | 34 | ns | 17 | 34 | ns |
|  | Ortho | 1,739.01 | 5.5014 | 0.0639 | moderate | 1 | 11 | ns | 1 | 27 | ns | 11 | 27 | ns |
| Adjusted cost per bed day  (Inpatient cost centre) | Gen | 1,489.73 | 5.9838 | 0.0502 | moderate | 3 | 13 | ns | 3 | 33 | ns | 13 | 33 | * |
|  | Med | 1,341.27 | 7.3212 | 0.0257 | large | 1 | 9 | ns | 1 | 29 | ns | 9 | 29 | * |
|  | OBG | 1,239.41 | 2.8933 | 0.235 | small | 4 | 17 | ns | 4 | 34 | ns | 17 | 34 | ns |
|  | Ortho | 1,319.10 | 7.4078 | 0.0246 | moderate | 1 | 11 | ns | 1 | 27 | ns | 11 | 27 | * |

**Supplementary material: Table S5 Unadjusted unit costs by state and type of provider (INR)**
